# Conservative treatment of fractures of the clavicle

**DOI:** 10.1186/1756-0500-4-333

**Published:** 2011-09-08

**Authors:** Silvana De Giorgi, Angela Notarnicola, Silvio Tafuri, Giuseppe Solarino, Lorenzo Moretti, Biagio Moretti

**Affiliations:** 1 Department of Clinical Methodology and Medical-Surgical Technologies - Orthopaedics Section, University General Hospital, Piazza G.Cesare 11, 70124 Bari, Italy; 2 Department of Biomedical Sciences-Hygiene Section, University General Hospital, Piazza G.Cesare 11, 70124 Bari, Italy

**Keywords:** Fracture, clavicle, shortening, conservative treatment

## Abstract

**Background:**

In the treatment of clavicle fractures, the choice of procedure depends on the possibility of restoring the anatomical functional integrity of the shoulder.

**Methods:**

We examined 71 patients (51 males and 20 females, mean age 38.9 years) who were affected by clavicle fracture sequelae. Demographic and clinical data and the site of the lesion were recorded for each partecipant. The dissatisfaction of the patient was determined by the presence of 1 or more affirmative answers on the Simple Shoulder Test. The Constant Shoulder Score was also included in the functional and clinical exams. We measured the length of the healthy clavicle and the previously fractured clavicle, and we expressed the difference in length in mm and in percentage shortening. We then examined the correlations between the shortening of the bone and the clinical and functional outcomes of the patients.

**Results:**

Sixty patients had a lesion of the diaphysis, 8 patients had a lesion of the lateral third of the clavicle, and 3 patients had a lesion of the medial third of the clavicle. The mean Constant Shoulder Score was 77.9, and 51 of the 71 patients were satisfied with their treatment. Radiography showed a mean clavicle shortening of 10 mm (mean percentage 6.5%). In the 20 dissatisfied patients, the mean clavicle shortening was 15.2 mm (9.7%). In these patients, we found a highly significant association between dissatisfaction with treatment and the amount of bone shortening, (p < 0.0001), as well as with a diaphyseal location (p < 0.05) and with the female sex (p = 0.004). No other variable related to the patient, the type of treatment or the fracture characteristics correlated with the treatment outcome.

**Conclusions:**

In the literature, measurements of the shortening of the bone segment following a fracture range between 15 and 23 mm, and marked shortening is correlated with the failure of conservative treatment. However, these data need to be reinterpreted in light of the physiological variability of the clavicle length, which ranges from 140 to 158 mm in the healthy population. Shortening of the bone by more than 9.7% should be the cut-off for predicting failure of conservative treatment.

## Background

Optimal shoulder function is achieved by the interaction of four joints (sternoclavicular, acromio-clavicular, scapulothoracic and glenohumeral) working in biomechanical harmony. It is impossible to intervene at one joint without affecting the efficiency of the other three [1]. The first two joints directly involve the clavicle, a long S-shaped bone with a mean length of 149 ± 9.1 mm that appears slender and less resistant in the central portion because it is not stabilised by ligaments or protected by musculotendinous structures [2].

Fractures of the clavicle are common, comprising up to 5% of all skeletal lesions in adults [3]. Clavicle fractures are more prevalent in young men and in older women [4]. In 69-82% of patients, they are localised at the level of the midshaft-diaphyseal third [5]. The most frequent mechanism of injury was a fall (39.6%), and coexisting injuries were found in 12.9% of patients [4]. In 73% of cases, dislocation of the end of clavicle occurs due to the actions of the sternocleiodomastoid muscle, which displaces the medial fragment superiorly and posteriorly, and of the deltoid and great pectoral muscles, which shift the lateral fragment inferiorly and anteriorly. These shifts cause a malaligned fracture with a superimposition of the two fragments that results in the shortening of the bone segment [6]. Conservatively treated fractures united in 96.9% of cases, and the time to union was not different when treated with a sling, a collar and cuff or figure-of-eight bandage [4,7,8]. In 5% of patients, these lesions lead to pseudoarthrosis, and this incidence is significantly increased in cases where the dislocation is more severe [9].

Mean post-traumatic shortening the fractured clavicle is approximately 1.2 cm, but a reduction of up to 3 cm has been described [10]. In the last decade, many studies have reported that a shortened clavicle can lead to pain, loss of strength, rapid fatigue, hyperesthesia of the hand and arm, difficulty sleeping on the affected side and aesthetic complications [11]. Many authors have observed the onset of pain, the degree of which is related to the extent of shortening of the bone segment; however, discordant lengths ranging from 1.5 to 2.3 cm have been reported [12]. For this reason, we elected to study the percentage of reduction of the bone segment length as compared to the pre-trauma length (or to the contralateral clavicle) to negate the constitutional physiological differences in clavicle length that are present in the population.

The aim of our work was to evaluate shoulder function and patient satisfaction in relation to the shortening of the fractured segment in patients suffering a clavicle fracture that was treated conservatively with the figure-of-eight bandage.

## Methods

This clinical retrospective study included patients who were undergoing conservative treatment with a figure-of-eight bandage for a clavicle fracture during the period from 2004 to 2010 at our Operative Unit of Orthopaedics and Traumatology. All of the patients received information about the aims of the study and signed an informed consent form prior to undergoing the clinical and diagnostic tests that were included in the study protocol. The study was authorised by the Ethics Committee of the General Hospital of Bari and was performed in accordance with the ethical standards of the 1964 Declaration of Helsinki.

Inclusion criteria were as follows:

- a blunt, uncomplicated clavicle fracture with a single focus, which was treated within three days of diagnosis

- conservative treatment with a figure-of-eight bandage (FEB)

Exclusion criteria were as follows:

- bilateral clavicle fractures

- a previous clavicle fracture or pseudoarthrosis

- an exposed clavicle fracture or associated fracture of the coracoid, an acromioclavicular or sterno-clavicular luxation, floating shoulders, lesions of the plexus and concomitant fractures in other sites

- the presence of disease impairing the function of either shoulder

Ninety-three partecipants met the inclusion criteria. Twenty-two patients decided not to participate because of travel or the time that was required to attend the clinical exam; therefore, a total of 71 participant participated in this study.

All of the patients were clinically reviewed, and the following were considered:

- demographic (age, sex) and clinical data (dominant limb, type of fracture) and history (fracture characteristics, months since the trauma occurred)

- the site of the lesion (medial, diaphysis or lateral) according to the 1988 Edinburgh classification [5] in which the clavicle is subdivided into three segments, a medial type (medial 1/5, the clavicle area lying medial to a vertical line drawn upward from the centre of the first rib), a diaphyseal type (middle 3/5ths) and a lateral type (lateral 1/5th, lateral to a vertical line drawn upward from the centre of the base of the coracoid process, a point normally marked by the conoid tuberosity)

- a self-administered questionnaire defining one or more affirmative answers on the Simple Shoulder Test as unsatisfactory, which verifies the presence of resting pain, aesthetic deformity, pain when leaning on the affected shoulder, differences in the range of movement and force as compared to the contralateral side and the loss of functional autonomy of the upper limb in daily activities [3,10-13]

- the Constant Shoulder Score, which assesses pain, degree of function, ROM and muscular force [14]

- an AP chest X-ray with a specific clavicle projection that is obtained by cephalic angling of the tube at 45° to avoid superimposition of the clavicle and ribs at a 1:1 scale [15]

Successful consolidation of the fracture was determined by the formation of a bone callus and the presence of a trabecular bridge over the fracture gap at the periosteal and endosteal level within 6 months of the trauma [16]. After calculating the length of the healthy segment and the affected segment by drawing a straight line through the medial point of the sternal and acromial borders, the difference was expressed in mm, and the percentage of shortening was quantified [15].

Quantitative variables were expressed as means and standard deviations (SD); categorical variables were expressed as frequency distributions. A t-test was used to compare the age difference between male and female patients, the Simple Shoulder Test results and the difference in follow-up time between satisfied and dissatisfied patients. The Wilcoxon test was used to compare the age and the Constant Scores of satisfied and dissatisfied patients. To assess the differences in frequency distributions, the Chi-square test was employed. To reveal correlations between shortening of the limb (percentage and absolute values), a logistic regression model was applied to the Constant Scores and R2 was calculated. A multivariate logistic regression was used to analyse factors that were associated with satisfied patients.

Significance was set at p < 0.05; Fisher Exact test was used when appropriate. Data processing was performed with Epi-Info 6.00 software (public domain software - CDC Atlanta, Georgia; WHO Geneva, Switzerland).

## Results

A total of 71 eligible patients who were affected by clavicle fractures and were treated within 72 hours of the trauma were recruited. All of the patients had been treated with FEB for a mean of 28 days (range 26-42 days, SD = 4 days) and had done hanging-type exercises and active and passive joint ROM for two weeks after the removal of the bandage. No patient presented with pseudoarthrosis complications, lesions of the brachial plexus or thoracic outlet syndrome.

The study sample included 51 males (71.8%) and 20 females (28.2%). The mean age was 38.9 years (SD = 13.3), 37.2 years (SD = 10.9) for females and 39.6 years (SD = 14.1) for males (t = 0.7; p > 0.05). The fracture occurred on the clavicle of the dominant arm in 47.9% of the patients (n = 34), 45.1% of the males (n = 23/51) and 55% (n = 11/20) of the females (0.5644; p = 0.452).

In 59 patients, the lesion was at the level of the diaphysis (83.1%). Three (4.2%) patients had a lesion of the medial site, and 9 (12.7%) patients had a lesion of the lateral segment. There was not a significant difference in the distribution of the fracture site between genders (Table 1; chi-square = 3.95; p = 0.14).

**Table 1:** Distribution of the fracture site for each gender

Site	Female		Male		Total	
	n	%	n	%	n	%
**Diaphyseal third**	14	70	45	88.2	59	83.1
**Lateral**	5	25	4	7.8	9	12.7
**Medial**	1	5	2	4	3	4.2
**Total**	20	-	51	-	71	-

When inquiring about physical exercise, 31 patients (43.7%) declared that they did heavy physical work or a considerable amount of sport, 22 (30.9%) led a sedentary life, while no type of physical activity was recorded for 18 patients (25.3%).

The mean follow up time was 32.7 months (SD = 17.2; range = 12-72) with no significant difference between males and females (t = 0.01; p = 0.99). The mean Constant Score was 77.9 (SD = 8.5; range = 50-90) and was lower in males (76.3, SD = 8.4) than in females (82.1, SD = 7.6; t = 2.65; p = 0.004). The mean clavicle shortening was 10 mm (SD = 5.0; range = 1-20), 10.7 mm (SD = 5.3) in males and 8.2 mm (SD = 3.7) in females (t = -1.9; p = 0.03); the mean percentage was 6.5% (SD = 3.1; range 0.6-12.2), with no significant difference between males (6.8%; SD = 3.3) and females (5.8%; SD = 2.5; t = -1.19; p = 0.12). Twenty-two patients (30.9%) had a shortening of the clavicle that was less than 5%, 39 patients (54.9%) had shortening between 5 and 10% and 10 patients (14.1%) had shortening greater than 10%.

According to the criteria described above for the Simple Shoulder Test, 51 partecipants (71.8%) were satisfied with the conservative treatment, and 21 (29.6%) patients were dissatisfied. There were no significant differences in follow up time (t = 0.01; p = 0.99) or in the mean age between dissatisfied and satisfied patients (H = 0.01; p = 0.9) (table 2).

**Table 2:** Patients who expressed satisfaction and dissatisfaction on the Simple Shoulder Test

Patient	Satisfied		Dissatisfied		Statistical analysis	p
	mean	%	mean	%		
**Cases**	51	71.8	21	29.6	-	-
**-**	**mean**	**DS**	**mean**	**SD**	-	-
**Follow up**	32.6	16.5	37.7	19.3	t = 0.01	p = 0.99
**Age (Months)**	39.2	14.8	38.9	12.8	H = 0.01	p = 0.9

A lower proportion of women (1/20; 5%) reported dissatisfaction as compared to men (19/51; 37.2%). For this comparison, Chi-square = 7.4 and Fisher exact p = 0.0004 (table 3).

**Table 3:** Gender of satisfied and dissatisfied patients

Gender	Satisfied		Dissatisfied	
	n	%	n	%
**Famale (total 20 cases)**	19	95	1	5
**Male (total 51 cases)**	32	62.8	19	37.2

Chi-square = 7.4; p = 0.0004

The proportion of dissatisfied patients did not significantly differ according to whether the fracture affected the dominant or non-dominant arm (Chi-square = 0.7; p = 0.4) (table 4). We also assessed the correlation between the level of satisfaction and the site of the lesion (Chi-square = 5.6; p = 0.05) (table 5).

**Table 4:** Proportion of satisfaction and dissatisfaction according to dominant and non-dominant arm

Epidemiological features	Satisfied		Dissatisfied	
	n	%	n	%
**Dominant arm (total 34 cases)**	8	23.5	26	76.5
**Non dominant arm (total 37 cases)**	12	32.4	25	67.6

Chi-square = 0.7; p = 0. 4

**Table 5:** Correlation between the degree of satisfaction and the site of the lesion in patients undergoing conservative treatment of clavicle fractures

Site of fracture	Satisfied		Dissatisfied	
	n	%	n	%
**Diaphyseal third**	39	66.1	20	33.9
**Medial**	9	100	0	-
**Lateral**	3	100	0	-

Chi-square = 5.6; p = 0.05

The percentage of dissatisfied patients was 25.6% among the patients with a percentage of clavicle shortening of 5-10% and 100% among the patients with a percentage of clavicle shortening that was greater than 10%; none of the patients with a clavicle shortening of less than 5% were dissatisfied (chi-square = 34.3; p < 0,00001).

**Figure 1:**
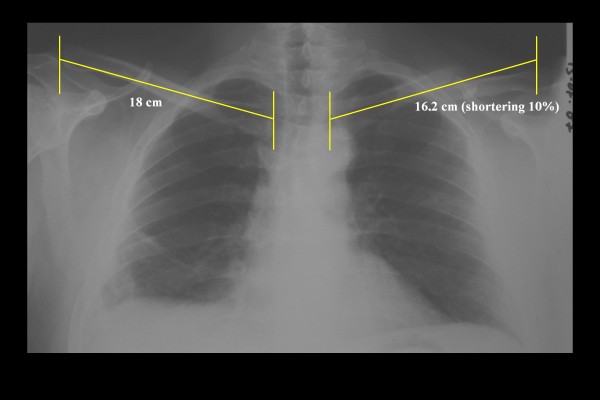
**Radiographic image of the clavicle of a dissatisfied patient after conservative treatment of a diaphyseal clavicle fracture**. The observed shortening was 18 mm or 10%; the Constant score was 65.

**Figure 2:**
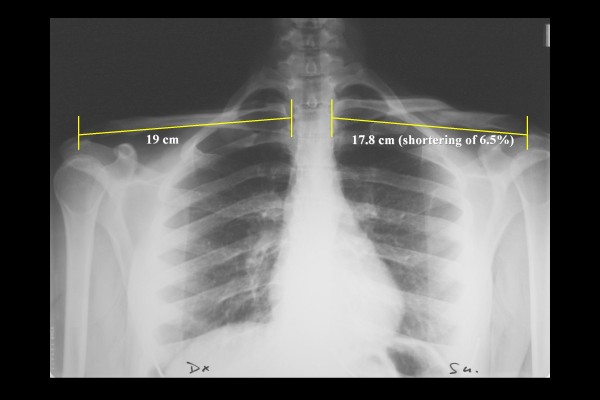
**Radiographic image of the clavicle of a satisfied patient after conservative treatment of a diaphyseal clavicle fracture**. The observed shortening was 12 mm, or 6.5%; the Constant score was 80.

In satisfied patients, the mean bone segment shortening was 7.9 mm (5.3%) versus 15.2 mm (9.7%) in patients who complained of functional dissatisfaction (Figures 1 and 2) (table 6).

**Table 6:** Mean shortening values expressed in mm and percentage variation in satisfied and dissatisfied patients

Mean shortening	Satisfied		Dissatisfied		t	p
	mean	SD	mean	SD		
**mm**	7.9	4.0	15.2	3.3	7.2	< 0.0001
**percentage**	5.3	2.6	9.7	1.6	7.2	< 0.0001

We also performed a multivariate logistic regression analysis using "patient satisfied" as the dependent variable (table 7).

**Table 7:** Factors associated with the "patient satisfaction" dependent variable in a multivariate logistic regression analysis

	Coefficient	t	P > t	[95% Conf. Interval]	
**Male sex**	-0.04	-0.62	0.539	-0.17	0.09
**% of clavicle shortening < 5%**	0.43	3.63	0.001	0.19	0.66
**% of clavicle shortening 5-10%**	0.31	3.07	0.003	0.10	0.50
**Diaphyseal third site**	-0.16	-1.13	0.263	-0.44	0.12
**Lateral site**	-.06	-0.38	0.702	-.38	0.26
**Dominant arm**	0.06	1.04	0.302	-0.05	0.17
**Costant score**	0.03	7.47	0.000	0.02	0.04

**Figure 3:**
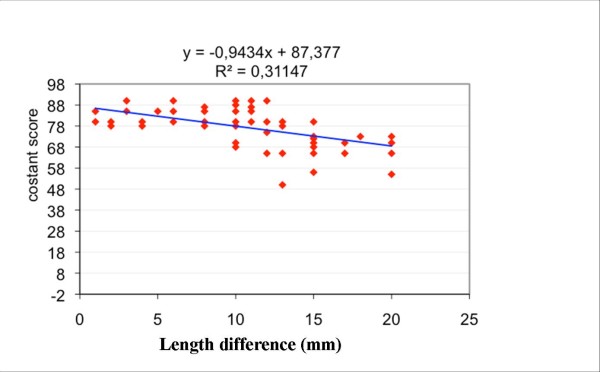
**The graph shows the correlation between clavicle shortening (expressed in mm) and the Constant Score**.

**Figure 4:**
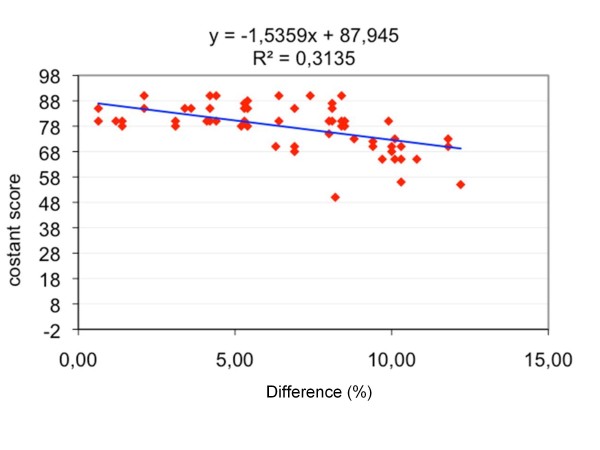
**The graph shows the correlation between clavicle shortening (expressed as percentage variation) and the Constant Score**.

The Constant Score was correlated with the shortening value expressed both in mm (r2 = 0.31; p < 0.001; Figure 3) and as a percentage (r2 = 0.31; p < 0.001; Figure 4). The mean Constant Score was 82.3 (SD = 4.0) in satisfied patients versus 66.6 (SD = 6.3) in dissatisfied patients (H = 44.5; p < 0.0001) (table 8).

**Table 8:** Constant Score in satisfied and dissatisfied patients

Constant Score	Satisfied		Dissatisfied		H	p
	mean	SD	mean	SD		
**score**	82.3	4.0	66.6	6.3	44.5	< 0.0001

## Discussion

The aim of this study was to assess the correlation between unsuccessful outcomes following conservative treatment of a clavicle fracture and the final length of the bone segment in absolute terms and as a percentage. Clavicle fractures are generally treated conservatively because this bone has excellent powers of repair that guarantee a good final consolidation of the lesion. Conservative treatment consists of the application of a figure-of-eight bandage (FEB) or a triangular bandage to restore the retro-positioning of the shoulder, resolving the superimposition of the stumps and limiting clavicular shortening. Surgery is usually reserved for the treatment of exposed lesions or highly displaced fractures to stabilise the bone malalignment with the use of screws, plates, endomedullary wires or bands [6].

Previous studies have analysed the risk of dysfunction after conservative treatment, which can be due to severe shortening of the bone segment, residual bone deformity, loss of force and persistent pain [6,9]. For these reasons, it is important to be able to distinguish the type of lesion and the type of patient that will likely benefit from conservative treatment.

Some studies have observed fewer cases of consolidation defects after surgery (2.2%) as compared to conservative therapy (15.1%) [17], whereas others reported a 37% risk of adverse events after a surgical procedure due to invasion of the periosteal structures that can lead to nerve damage in the bone shafts, blood loss and post-traumatic hematoma, which can delay healing [14]. Postoperatively, approximately 6% of patients suffer the onset of osteomyelitis or infections of the soft tissues or surgical scar [18], and this risk is increased if the patient has a second operation to remove metal fixation devices [19]. Some patients report discomfort due to metal fixation devices, which can often be felt through the soft tissues [20]. Others complain of an aesthetically displeasing scar [21-23]. After removal of the various possible devices, the loss of the protective stabilising effect poses a risk of re-fracture in approximately 8% of cases [24].

According to the literature, the incidence of failure of conservative treatment of clavicle fractures ranges from 4.4% to 31% in terms of pain, loss of force, rapid fatigue, paresthesia, pain when lying on the affected shoulder and aesthetic defects [3,10,25]. In our case series, these symptoms were observed in 21 patients, corresponding to 29.6% of the cases studied. In agreement with literature reports [5], we found a correlation between the onset of shoulder dysfunction and a diaphyseal rather than a medial or lateral fracture site. This may depend on the anatomical characteristics of the bone. The medial end is convex and the lateral end is concave, whereas the diaphysis, or midshaft, is tubular, thinner, has a lesser medullary component, is subject to more twisting forces and has a lesser repair capacity [26].

Neer suggests a statistical association between the degree of shortening of the bone segment and poor clinical results, with an increased risk of evolution to pseudoarthrosis [27]; in our study, the mean reduction of the clavicular length in dissatisfied patients was 15.2 mm, but discordant data are reported in literature. Eskola et al. identified 15 mm as the threshold value above which pain was likely to be present [7], whereas Hill et al. reported unsatisfactory results with a bone shortening of more than 20 mm [11] but underlined that this situation is not certain to lead to pseudoarthrosis. Postacchini et al. determined the cut-off for surgical treatment as a bone length reduction of more than 2.3 cm [24]. However, these different values do not take into account the constitutional variations in clavicle length that are present in the population. In fact, we believe that a 2 cm reduction in length of a long clavicle bone will be better compensated for than the same reduction in a short bone. For this reason, rather than adopting an absolute shortening value as previously done in the literature, we have calculated the percentage shortening value as compared to the original length. We found a correlation between a reduction by more than 9.7% and the onset of scapulohumeral dysfunction as demonstrated by a lower Constant Score. In their recent work, Postacchini et al. also recognised the utility of the percentage reduction value to assess the prognosis of conservative treatment of clavicle fractures [28]. They observed a greater statistical incidence of pseudoarthrosis in cases with a bone shortening exceeding 15%. This is supported by our finding of greater patient dissatisfaction with functional outcomes in cases with bone segment shortening of more than 9.7%.

There is still no consensus in literature as to whether conservative treatment of clavicle fractures is the optimal treatment in most cases [29] or if surgical indications should be extended [30]. Hillen et al. noted that there is still debate about which patients should be candidates for surgical bone synthesis but suggested that in cases involving severe dislocations, comminuted fractures, severe high energy trauma, involvement of the dominant limb, young subjects or sportsmen needing rapid, complete recovery and women and elderly patients, there is a high risk of failure after conservative treatment [31].

The results of the present study demonstrate poorer outcomes when the fracture occurs at the midshaft and when the shortening of the bone segment is more than 9.7% as compared to the original length. We also observed a greater degree of dissatisfaction in male patients.

However, our study has limitations. The study sample is relatively small, and there is no surgical control group. The Constant Score application at heterogeneous follow up points diminished its utility. Because this was a retrospective study, no functional data about the pre-treatment Constant Score were available. This data could have served to calculate the post-treatment improvement and better quantified each patient's recovery. Another limitation is that the data received a post hoc analysis.

Moreover, our results have been interpreted in the context of those reported by other authors who used different criteria for assessing the degree of patient satisfaction. Finally, AP chest radiography is unable to study shifts of the bone stumps in the sagittal plane; imaging of this type of shift requires a CT scan. It would be useful to design a prospective study with a specified minimal follow up time for administration of the survey to improve the results of our research.

## Conclusions

While conservative treatment remains the gold standard for minimally displaced clavicle fractures, in cases with severe dislocation of the focus, surgery may be indicated, depending on the clinical-instrumental characteristics of the case. The present study assessed the reliability of using the percentage shortening of the bone segment as a means of predicting the failure of conservative treatment of a clavicle fracture. Although our results cannot be generalised, the validity of basing the therapeutic decision on the percentage shortening value as compared to the initial length of the segment could be validated in multi-centric studies using larger population samples.

## List of abbreviations

FEB: figure of eight bandage; SD: standard deviation; CT: computed tomography.

## Competing interests

The authors declare that they have no competing interests.

## Authors' contributions

DGS and NA gave substantial contributes in the drafting the manuscript and in the revising it for the intellectual content. SG and ML participated in the acquisition of data of case reports. TS participated in the design of the study and performed the statistical analysis. MB participated in the analysis and interpretation of data, and reviewed the manuscript. All authors read and approved the final manuscript.
